# Supplementation with Queen Bee Larva Powder Extended the Longevity of *Caenorhabditis elegans*

**DOI:** 10.3390/nu14193976

**Published:** 2022-09-24

**Authors:** Tong Zhao, Liming Wu, Fangfang Fan, Yaning Yang, Xiaofeng Xue

**Affiliations:** 1 Institute of Apicultural Research, Chinese Academy of Agricultural Sciences, Beijing 100093, China; 2 Lao Dian Huang Institute of Life Sciences, Suzhou 215000, China; 3 School of Ecology and Environment, Anhui Normal University, Wuhu 241000, China

**Keywords:** antiaging activity, freeze-dried queen bee larva powders, *Caenorhabditis elegans*, transcriptome analysis

## Abstract

Queen bee larva (QBL) is one kind of important edible insect that is harvested during royal jelly production process. QBL has many physiological functions; however, limited information is available regarding its antiaging effects. In this study, the antiaging function of freeze-dried QBL powder (QBLP) was investigated by combining the *Caenorhabditis elegans* (*C. elegans*) model and transcriptomics. The administration of QBLP to *C. elegans* was shown to improve lifespan parameters. Additionally, QBLP improved the mobility of nematodes. Transcriptome analysis showed the differentially expressed genes (DEGs) were significantly enriched in Gene Ontology (GO) terms that were almost all related to the biological functions of cell metabolism and stress, which are associated with lifespan. The Kyoto Encyclopedia of Genes and Genomes (KEGG) analysis suggested that the lifespan of *C. elegans* was related to the longevity regulating pathway-worm. The expression levels of the key genes *sod-3*, *gst-6*, *hsp-12.6*, *lips-7*, *ins-8*, and *lips-17* were upregulated. *sod-3*, *hsp-12.6*, *lips-7*, and *lips-17* are downstream targets of DAF-16, which is an important transcription factor related to lifespan extension. CF1038 (*daf-16(mu86)*) supplemented with QBLP did not show a life-prolonging. This indicates that the antiaging function of QBLP is closely related to *daf-16*. Thus, QBLP is a component that could potentially be used as a functional material to ameliorate aging and aging-related symptoms.

## 1. Introduction

Aging is a physiological process by which the functions of an organism progressively decline over time. Aging affects every organ in the body and promotes many diseases, including neurodegenerative disorders, cardiovascular diseases, pulmonary diseases, diabetes, and cancer [1,2]. In 2019, 9% of the global population was considered to be older (65+), and this is expected to increase to 16% by 2050; thus, the demand for antiaging health products to ameliorate age-related complications is also on the rise [3,4]. Therefore, in the past decade, research on the use of natural active ingredients to prevent aging and age-related diseases has received substantial attention, and these studies are of great significance for the in-depth analysis of the aging of individuals, promotion of human physical health, treatment of age-related diseases, and extension of the healthy lifespan of human beings [5].

Queen bee larva (QBL) is an important edible insect that is harvested for use as by-product of royal jelly production [6]. In contrast to worker larva, which is fed royal jelly for the first 3 days after hatching, QBL are exclusively fed royal jelly [7]. In the process of royal jelly production, QBL are removed and discarded. Nevertheless, some recent studies showed that QBL is rich in proteins, fatty acids, and essential amino acids [8,9]. Consequently, similar to other edible insects, QBL also has high nutritional value [10]. Numerous studies have reported the antiaging [11], antioxidant [12], antihypertensive [13], anticancer [14], antiobesity [15], and antidiabetic [16] activities of edible insects. In addition, QBL, which are well-known bee products, have been reported to exert beneficial effects on health. For example, QBL can ameliorate p-chlorophenylalanine-induced insomnia and insomnia-related neurotransmitter disorders [8]. However, there are few reports about the antiaging effects of QBL.

*Caenorhabditis elegans* (*C. elegans*), is a free-living nematode with a simple physiological structure. *C. elegans* has the characteristics of easy cultivation and a short life cycle. The genetic pathways of *C. elegans* are well known, and 60%-80% of the nematode genome sequences are homologous with the human genome sequence [17]. Based on these advantages, *C. elegans* has been widely used to evaluate the antiaging properties of substances [18,19]. In *C. elegans*, signaling pathways that regulate aging, including the insulin/insulin-like growth factor signaling (IIS) pathway, SKN1/Nrf2 pathway and mitochondrial electron transport chain pathway, have been studied extensively [20,21]. López-García et al. found that two plant sterol-enriched fruit beverages extended the mean lifespan of nematodes through *daf-16* and probably through the inhibition of the insulin-IGF-1 pathway [22]. The results of Deng et al. suggested that *Schizophyllum commune* (*S. commune*)-fermented supernatant supplemented with Radix puerariae could significantly prolong the lifespan and healthspan of *C. elegans*, and these effects are mainly related to the IIS pathway, SKN1/Nrf2 pathway and mitochondrial metabolism pathway [23].

The present study proposed the use of *C. elegans* as the model system for investigating the antiaging effects of QBL. The antiaging capacity of freeze-dried QBL powder (QBLP) was evaluated by lifespan and locomotion assays, and transcriptome analysis was used to further understand the metabolic targets of QBLP. This study advances our understanding of the antiaging capacity of QBL and provides new insights into the potential health benefits of QBL treatment based on transcriptome profiling.

## 2. Materials and Methods

### 2.1. C. elegans Strains, Growth Conditions, and QBLP Treatment

The wild-type N2 strain and CF1038 (*daf-16(mu86)*) were selected for use in this experiment. All strains were cultured on nematode growth medium (NGM) inoculated with *Escherichia coli* OP50 strain at 20 °C [24]. All the strains were provided by Anhui Normal University (Wuhu, China). Synchronized worms were grown until the L4 stage. They were washed with M9 buffer and collected by centrifugation [25]. To prepare QBLP-supplemented food, appropriate amounts of QBLP (0.02, 0.2, and 2 g/mL) were dissolved in the NGM. Then, the collected worms were exposed to different concentrations of QBLP. QBLP was provided by Yunnan Laodianhuang Food Co., Ltd. (Kunming, China). Briefly, 4-day-old QBL was used for homogenization with JTM-60 Colloid Mill (Shenyang Xiangyang Colloid Mill Factory, Shenyang, China), and then the TS8606 Vacuum Freeze Dryer (Jiamao Wandeford Biotechnology Co., Ltd., Dongying, China) was used to dry QBL. The vacuum degree was 0.3 MPa. The temperature was 60 °C and lyophilized for 24 h. Finally, QBLP was obtained. Control was used in which no additional QBLP was added to the medium. Two positive controls were used in which 1 mM Nicotinamide mononucleotide (NMN) (Shanghai Yuanye Bio-Technology Co., Ltd., Shanghai, China) or 50 mM metformin hydrochloride (Shanghai Yuanye Bio-Technology Co., Ltd., Shanghai, China) was added to the medium.

### 2.2. C. elegans Lifespan Assay

After age synchronization, we transferred 40 young adult nematodes to fresh NGM plates. In addition, 2′-deoxy-5-fluorouridine (FUDR) (Sigma, St. Louis, MO, USA) was added to the plate to block *C. elegans* reproduction. QBLP was added to liquid NGM at three different final concentrations: 0.02, 0.2 and 2 g/L. Lifespan is monitored daily from the first day of adulthood. The numbers of live and dead worms were recorded every two days until all the worms were dead. The animals were considered to be dead when no pharyngeal pumping was observed or when the nematodes did not respond to touch. The mean and median lifespans were calculated. Three replicates were analyzed per exposure group [26,27].

### 2.3. C. elegans Locomotion Assay

*C. elegans* locomotion is characterized by sinusoidal movement, and it is an age-related parameter [28]. To determine the effect of QBLP on *C. elegans*, the nematodes were exposed to 0.02, 0.2, and 2 g/L QBLP, 1 mM NMN, or 50 mM metformin hydrochloride, respectively. Then, we analyzed 6-day-old, 9-day-old, and 12-day-old adult worms. To measure locomotion, age-synchronized nematodes (*n* = 30) were placed on NGM plates without OP50 and allowed to crawl for approximately 3 min to eliminate *Escherichia coli*. Then, each worm was incubated in M9 buffer with an acclimation time of 1 min [29]. Mobility was measured under a dissecting microscope by counting the number of body bends performed by the individual worm in 30 s [30]. Three replicate experiments were performed for each test dose.

### 2.4. RNA Extraction and Transcriptome Analysis

RNA for transcriptome sequencing was extracted from *C. elegans* treated with 0.2 g/L QBLP for 14 days and control-treated worms (no additional QBLP was added to the medium). About 2000 nematodes were pooled to generate a single biological sample, and RNA-seq library was constructed by three biological replicates. RNA extraction and transcriptome analysis were performed using Majorbio Medical Technology Corporation (Shanghai Majorbio Bio-pharm Technology Co., Ltd., Shanghai, China). All the transcriptome analyses were performed based on the high-quality clean data. Gene expression was calculated using the normalized expression values as FPKM by RSEM (v1.3.3, Bo Li and Colin Dewey, Cambridge and Madison, USA). The analysis of differential expression between groups was then performed using DEGSeq (v 1.38.0, Bioconductor, MA, USA). An adjusted *p*-value < 0.05 and absolute value of log_2_ fold change ≥ 1 were set as the thresholds for significantly differential expression of coding genes.

### 2.5. Functional Analyses

Blast2go (https://www.blast2go.com (accessed on 6 June 2022)) was used for Gene Ontology (GO) enrichment analysis of the differentially expressed genes (DEGs), and gene length bias was corrected. Kyoto Encyclopedia of Genes and Genomes (KEGG) is a database resource based on molecular-level information, especially large-scale molecular datasets generated by genome sequencing and other high-throughput experimental technologies, for understanding the high-level functions and utilities of biological systems, such as cells, organisms, and ecosystems (http://www.genome.jp/kegg (accessed on 14 April 2022)). We applied KOBAS software (v 2.1.1, Biological Information Center of Peking University, Beijing, China) to test the enrichment of DEGs in KEGG pathways.

### 2.6. Quantitative Real-Time PCR Validation

The expression of randomly selected DEGs was validated using quantitative real-time PCR (qRT–PCR) [31]. The primers were designed using Primer3plus software (v 3.2.6, EMBL, Heidelberg, Germany) (www.primer3plus.com (accessed on 6 March 2022)) and are listed in Supplementary Table S1. Total RNA for qRT–PCR was extracted from *C. elegans* treated with 0.2 g/L QBLP for 14 days and control-treated worms (no additional QBLP was added to the medium). The RNA concentration was measured using a NanoDrop 2000 (Thermo Fisher, Waltham, MA, USA). cDNA was generated using the Transscript One-Step gDNA Removal and cDNA Synthesis Supermix (Transgen, Beijing, China). qRT–PCR was performed using 2× TSINGKE Master qPCR Mix (SYBR Green Ⅰ) (Tsingke Biotechnology, Beijing, China) on a QuantStudio 1 Real-Time PCR system (Thermo Fisher A40425, Waltham, MA, USA) according to the instructions of the manufacturer. The quantitative RT–PCR (RT–qPCR) reactions consisted of 10 μL of 2× SYBR Green PCR Master Mix, 0.4 μL of each forward and reverse primer (Sangon Biotech, Shanghai, China), 1 μg of cDNA from samples and sterile distilled water, and the final volume was 20 μL. The mRNA abundance was normalized to that of the housekeeping gene β-actin. The RT–qPCR conditions were as follows: initial holding stage 95 °C for 30 s, followed by 40 cycles (95 °C for 5 s and 60 °C for 30 s); then, 95 °C for 15 s and 60 °C for 1 min; 95 °C for 1 s. Then, the relative expression was calculated according to the 2^−ΔΔCT^ method. Three technical replicates were set for each biological replicate [27].

### 2.7. Statistical Analysis

Lifespan curves were generated with Origin (OriginLab Inc., Northampton, MA, USA). Log-rank test was performed to analyze the statistical significance of lifespan changes using IBM SPSS software version 26.0 (International Business Machines Corporation, Amonk, NY, USA). The error bars represent SEMs. Multiple group comparisons were conducted using one-way analysis of variance (ANOVA).

## 3. Results

### 3.1. Effects of QBLP Supplementation on the Lifespan of C. elegans

**Figure 1:**
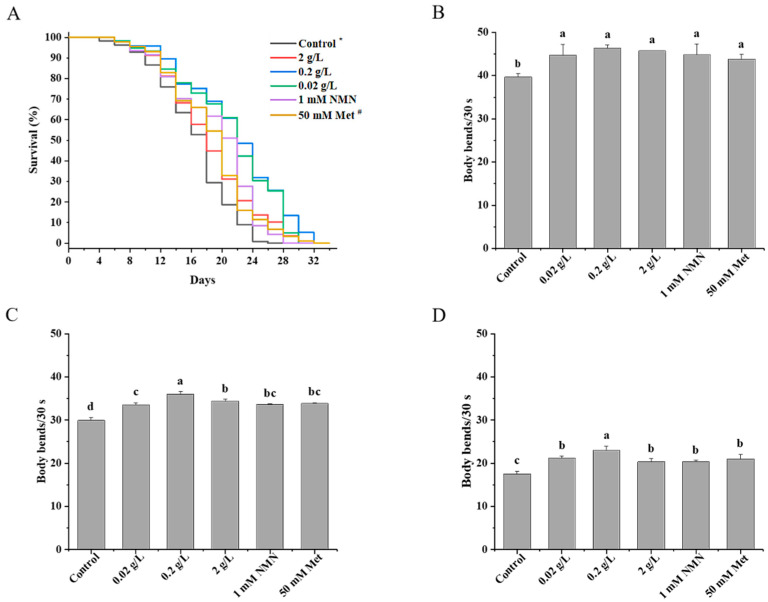
(**A**) Effect of different doses of QBLP on the lifespan of *C. elegans*. (**B**–**D**) Effect of different doses of QBLP for 6 days, 9 days, and 12 days on the locomotion of *C. elegans*. * Control indicates the control group (no additional QBLP was added to the medium). ^#^ Met indicates metformin hydrochloride. Different letters indicate significant differences between groups (*p* < 0.05). The data are the mean ± SEM.

Figure 1A shows that QBLP extended the lifespan of nematodes at all three concentrations tested compared with the control. When worms were exposed to concentrations of 0.02, 0.2, and 2 g/L QBLP, their mean lifespans were 20.3, 23.7, and 23.1 days, respectively. As the QBLP concentration increased, the mean lifespan of *C. elegans* increased by 9.7%, 28.1%, and 24.9%, respectively (Table 1). Among these treatments, treatment with 0.2 g/L QBLP had the most obvious effect on longevity. It is well known that the toxic effects of many natural substances used in therapy at high doses may overshadow their efficacy [32,33]. Consistent with this, we found that 2 mg/L supplementations of QBLP, higher than their optimum concentrations, did not show any pro-longevity effect. The effects of 0.2 g/L QBLP on nematode aging were significantly better than those of 1 mM NMN or 50 mM metformin hydrochloride.

### 3.2. Effects of QBLP Supplementation on the Locomotion of C. elegans

*C. elegans* exhibits multiple physiological phenotypic changes during senescence [34]. Motility is an important physiological and functional indicator of healthspan. As *C. elegans* ages, locomotion rates in liquid decline, and the severity of impairment is approximately correlated with the degree of muscle deterioration, which bears striking cell biological similarity to human sarcopenia [35]. We tested the effects of 0.02, 0.2, and 2 g/L QBLP supplementation on the decreased locomotion in aging worms at day 6 of life, day 9 of life, and day 12 of life. As shown in Figure 1B–D, the locomotion of nematodes decreased gradually from day 6 to day 12 as the nematodes aged. However, the number of body bends performed by worms exposed to 0.02, 0.2, and 2 g/L QBLP was significantly higher than that performed by control worms. These results showed QBLP could delay the decreased locomotion of *C. elegans* caused by aging, among them 0.2 g/L QBLP was the most effective concentration. Of 12-day-old worms in the 0.2 g/L QBLP group, the body bends of nematodes increased by 31.1%, while increased 20.2% and 16.9% in 9-day-old and 6-day-old worms, respectively. Notably, 0.2 g/L QBLP has a better effect on improving the locomotion of aging nematodes compared with 1 mM NMN or 50 mM metformin hydrochloride. These results are consistent with the lifespan assay.

### 3.3. Overview of RNA-Sequencing

In order to investigate the antiaging effects of QBLP, whole RNA was extracted from *C. elegans* treated with 0.2 g/L QBLP for 14 days and from control worms, and whole gene expression was evaluated by RNA-Seq. The sample names were as follows: C_14_1, C_14_2, and C_14_3 (control group); S_14_1, S_14_2, and S_14_3 (*C. elegans* treated with 0.2 g/L QBLP for 14 days); each sample was analysed in three biological replicates. The experiment generated 47716312-53119192 raw reads per sample. After quality control measures were adopted, 45854876-51084886 clean reads were obtained for each sample, and 96.49-97.12% of clean reads in each library were mapped onto the *C. elegans* reference genome. The Q30 ranged from 93.39 to 94.01% for each sample (Supplementary Table S2). The results revealed that genes were saturated irrespective of the control group or the treated groups. This sequencing amount can cover the majority of expressed genes.

### 3.4. Differential Expression mRNA Analysis

**Figure 2:**
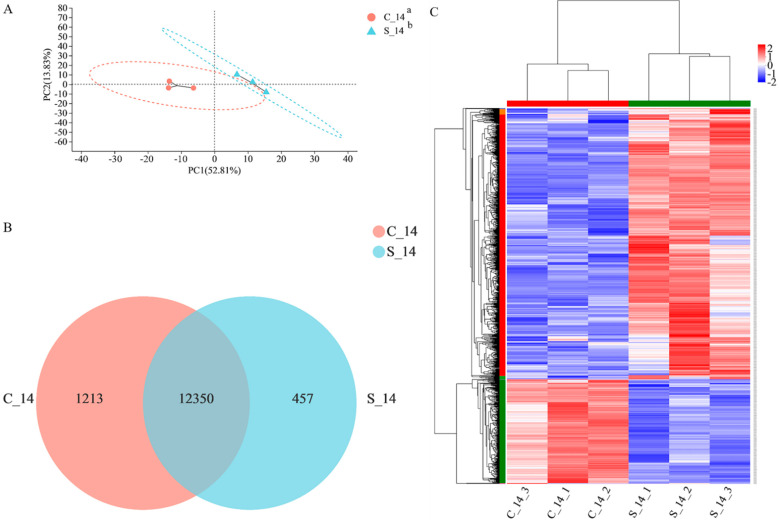
(**A**) Principal component analysis (PCA) based on mRNA expression. (**B**) Venn diagram illustrating DEGs in the QBLP treatment groups relative to the control group. (**C**) Heatmap of mRNA expression profiles. ^a^ C_14 indicates the control group (no additional QBLP was added to the medium). ^b^ S_14 indicates the treatment group (*C. elegans* treated with 0.2 g/L QBLP for 14 days).

To further explore the regulatory effect of QBLP on *C. elegans*, we compared and analyzed the *C. elegans* groups treated with or without QBLP. The overall distribution is shown in Figure 2. Principal component analysis (PCA) was used to assess the distance association among the six samples according to the changes in expression. The PCA score plot indicated a clear separation between the two groups (Figure 2A). Gene expression levels of RNA-Seq data were calculated by RSEM, and 13,563 and 12,807 mRNAs were identified in the control and treatment groups, respectively (Figure 2B). Then, to understand the mRNA expression patterns in the *C. elegans* samples, we generated a heatmap using hierarchical clustering analyses to show the different changes in mRNA expression (Figure 2C). Red indicates genes with high expression, and blue indicates genes with low expression. DEGs were selected as the thresholds with absolute value of log_2_ fold change ≥ 1 and *t*-test *p* values < 0.05, and the results showed significant changes in gene expression between control and treated *C. elegans*. We found 1049 DEGs after comparing the two groups, which included 758 upregulated DEGs and 291 downregulated DEGs (Supplementary Table S3). These genes may be important genes that are regulated by QBLP in *C. elegans*. 

### 3.5. Gene Ontology Functional Analysis of mRNAs

**Figure 3:**
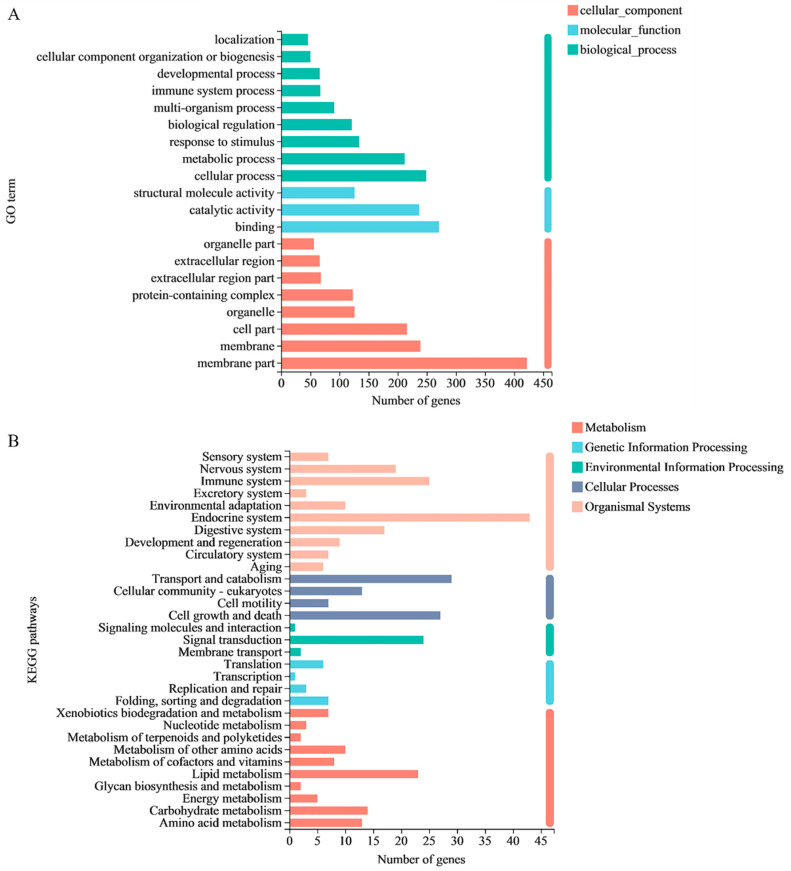
(**A**) Top 20 GO terms based on the transcriptome of QBLP-treated *C. elegans* relative to that of control worms. (**B**) Top pathways that were altered after QBLP treatment in *C. elegans*.

To gain a deeper understanding of the regulation of gene expression during the aging transitions of *C. elegans*, especially at the biological process (BP), molecular function (MF), and cellular component (CC) levels, we aggregated GO annotations for these DEGs. Here, 44 enriched GO terms were identified in three categories: 11 in molecular function, 15 in a cellular component, and 18 in biological process (Supplementary Table S4). As shown in Figure 3A, based on BPs in the GO classification, the DEGs were classified into different functional categories, and the top three enriched GO terms were “cellular process”, “metabolic process”, and “response to stimulus”. Together, these results indicate that QBLP treatment likely affects the cellular metabolism of nematodes. In the CC domain, the top three GO terms were “membrane part”, “membrane”, and “cell part”. In the MF, “binding”, “catalytic activity”, and “structural molecule activity” were the most highly represented terms. These significantly enriched GO terms were almost all related to the biological functions of cell metabolism and stress. Cell metabolism and stressful stimuli are associated with lifespan [36,37].

### 3.6. KEGG Pathway Analysis of mRNAs

In order to further understand the biological function of the DEGs between the two groups, KEGG enrichment analyses of the mRNAs in the two groups were performed. In this study, associations with human disease pathways were excluded. Based on KEGG pathway mapping, the DEGs were classified into 177 KEGG functional categories. A summary of the findings is presented in Figure 3B and Supplementary Table S5. An interesting observation is that six DEGs directly affect aging, and all are related to the longevity regulating pathway-worm. Supplementary Table S6 shows the 6 DEGs that participated in the longevity regulating pathway-worm. Thus, the longevity regulating pathway-worm was the main pathway that affects aging [38]. *sod-3*, *gst-6*, *hsp-12.6*, *lips-7*, and *lips-17* are downstream of DAF-16. DAF-16 is indispensable both for lifespan regulation and stress resistance [39]. This result is consistent with the GO analysis.

### 3.7. Validation of mRNA Expression

**Figure 4:**
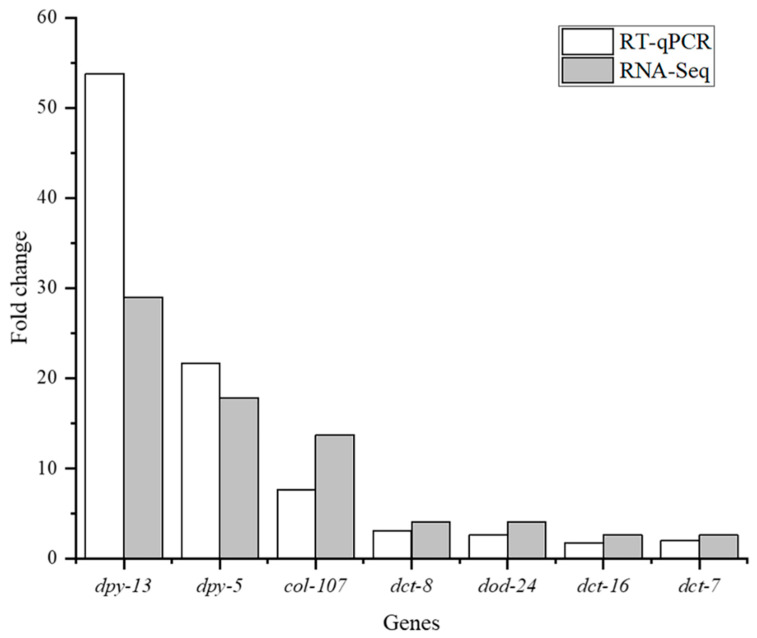
Validation of RNA-Seq data through RT–qPCR analysis of selected genes in *C. elegans* treated with QBLP for 14 days. Relative mRNA expression (calculated based on 2^−ΔΔCT^) of *dpy-13*, *dpy-5*, *col-107*, *dct-8*, *dod-24*, *dct-16*, and *dct-7*, with *β-actin* as the reference gene.

To verify the RNA-Seq results, we confirmed the differential expression of 7 selected genes (*dpy-5*, *dpy-13*, *col-107*, *dct-8*, *dod-24*, *dct-16*, and *dct-7*) by RT–qPCR analysis. Overall, the RT–qPCR results indicated that the expression trends of all selected genes were consistent with the RNA-Seq data (Figure 4), proving the reliability of the transcriptome analysis.

### 3.8. Effects of QBLP Supplementation on the Lifespan of daf-16 Mutants

**Figure 5:**
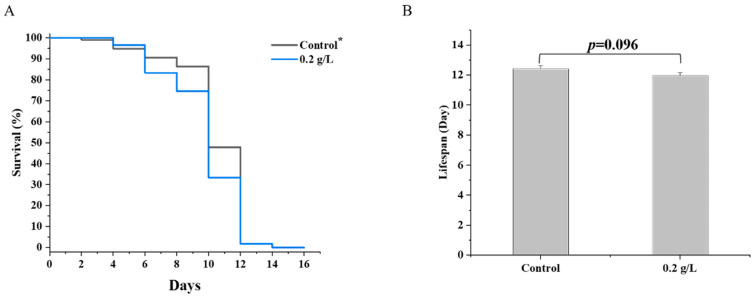
(**A**) Effect of QBLP on the lifespan of mutant worms CF1038 (*daf-16 (mu86)*). (**B**) Mean lifespan results for mutant worms CF1038 (*daf-16 (mu86)*) treated with 0.2 g/L of QBLP. * Control indicates the control group (no additional QBLP was added to the medium).

To more thoroughly investigate whether supplementary QBLP extends *C. elegans* lifespan by *daf-16*, we performed lifespan assays of *daf-16* mutants by CF1038 animals. The mean lifespan of the control group and 0.2 g/L QBLP supplementary group were 12.4 and 12.0 days, respectively. There was no increase in lifespan produced by QBLP in the *daf-16* mutant CF1038 (Figure 5 and Supplementary Table S8), while QBLP can prolong the lifespan of wild-type nematodes (Figure 1A and Table 1), an indication that the antiaging effect of QBLP is dependent on *daf-16*.

## 4. Discussion

Aging manifests as time-dependent functional decline and increased mortality, and it ultimately results in diseases and death in most living organisms. Thus, the development and utilization of food resources with potential antiaging activities have attracted increasing attention from researchers [40]. *C. elegans* is a convenient and well-established experimental model, and it is a popular model that is used in aging research because it shares many genes with humans and its lifespan is very short; thus, this model allows scientists to quickly assess the effects of genetic and environmental interventions to extend healthy lifespan [41].

In this study, we used three different doses of QBLP (0.02, 0.2, and 2 g/L) to test its functional effects on the lifespan and motility of *C. elegans*. Figure 1 and Table 1 show that, at all concentrations, the studied food improved the lifespan and healthspan parameters of the *C. elegans* model organism. NMN and metformin hydrochloride are commonly prescribed antiaging medications. Compared with 1 mM NMN and 50 mM metformin hydrochloride, 0.2 g/L QBLP more effectively improved the lifespan and healthspan of *C. elegans*. Therefore, QBLP can effectively prevent aging in *C. elegans* and reduce the decline in locomotion caused by aging.

However, the mechanism by which QBLP prolongs lifespan in *C. elegans* remains unclear. Nematode lifespan is known to be regulated by multiple classical signaling pathways, including the sirtuin 1 signaling pathway, insulin/insulin-like growth factor (IIS) pathway, and target of rapamycin (TOR) signaling pathway [42]. Next, we investigated changes in the transcriptomes between *C. elegans* grown on control NGM and those grown on NGM supplemented with 0.2 g/L QBLP. The analysis found that 1049 mRNAs were differentially expressed between the two groups (Figure 2C). Therefore, GO functional annotation and KEGG pathway analysis used DEGs.

In function analyses, the significantly enriched pathways in *C. elegans* supplemented QBLP related to endocrine system, transport and catabolism, cell growth and death, and aging. Specific include lysosome (map04142), platelet activation (map04611), apoptosis (map04210), autophagy-animal (map04140), longevity regulating pathway-worm (map04212). The pathway of longevity regulating pathway-worm is directly related to aging. For longevity regulating pathway-worm, six DEGs (*sod-3*, *gst-6*, *hsp-12.6*, *lips-7*, *ins-8*, and *lips-17*) were involved and all of them were notably upregulated (Supplementary Table S6). *sod-3*, *gst-6*, *hsp-12.6*, *lips-7*, and *lips-17* are downstream of DAF-16. Many DAF-16 target genes have been reported to control aging in *C. elegans*, including *sod-3*, *hsp-12.6*, *lips-7*, and *lips-17* [43,44,45,46,47,48,49]. Considering that there may be many genes not included in the KEGG database, this study further searched for genes related to DAF-16 among DEGs and another 7 genes (*dod-22*, *dod-17*, *dct-8*, *dod-24*, *dod-3*, *dct-16*, and *dct-7*) were discovered (Supplementary Table S7). Of these, *dod-22*, *dod-17*, *dod-24*, and *dod-3* are downstream of DAF-16. Collectively, the antiaging effect of QBLP on *C. elegans* is related to DAF-16. The loss of lifespan extension effect produced by QBLP in *C. elegans* CF1038, a *daf-16* loss-of-function mutant strain, further supports this conclusion.

As a naturally functional food, QBLP is a unique mixture. Given that approximately 50% of the dry mass of QBL is crude proteins, polypeptides and proteins in QBL were identified as the most significant bioactive components [50]. Wang et al. has emphasized that healthy proteostasis contributes to healthy aging, and that DAF-16 contributes in part to lifespan by promoting healthy proteostasis [51]. Furthermore, several studies have revealed the interplay of diet-based interventions for aging. Royal jelly-mediated longevity and stress resistance in *C. elegans* may be regulated by the interaction of DAF-16, SIR-2.1, HCF-1, and 14-3-3 proteins [33]. This suggests that QBLP supplementation may trigger a sophisticated regulatory mechanism, to thereby promote pro-longevity in *C. elegans*. Taken together, the active ingredients responsible for the antiaging function of QBLP are not clear and warrant further investigation.

Overall, QBLP can effectively exhibit potent antiaging activity in *C. elegans*. Further, GO and KEGG analyses revealed that *C. elegans* treated with 0.2 g/L QBLP for 14 days may exhibit changes in their biological functions related to cell metabolism and stress. In addition, QBLP can increase the lifespan due to the activation of DAF-16. Many other pathways affect the lifespan of nematodes, such as lysosome [52], apoptosis [53], autophagy-animal [54], which are worthy of further verification. Further experiments are needed to investigate whether these effects also occur in mammalian species, such as mice and humans; such studies will increase the possibility of developing QBLP as a novel antiaging agent. We hope our results will be helpful for the development of new QBL functional products.

## Figures and Tables

**Table 1:** Effect of QBLP supplementation on the lifespans.

**Condition**	**Number of Animals that Died/Total**	**Mean Lifespan** **(Days ± SEM)**	**Median Lifespan (Days ± SEM)**	**Change from Control (d)**	**Increase versus Control (%)**
Control ^a^	112/120	18.5 ± 0.5	20.0 ± 0.6	/	/
0.02 g/L	116/120	20.3 ± 0.5 **	20.0 ± 0.7	1.8	9.7
0.2 g/L	97/120	23.7 ± 0.7 ***	24.0 ± 0.7	5.2	28.1
2 g/L	118/120	23.1 ± 0.6 ***	24.0 ± 0.5	4.6	24.9
1 mM NMN	94/120	21.1 ± 0.6 ***	24.0 ± 0.5	2.6	14.1
50 mM Met ^b^	88/120	20.6 ± 0.6 ***	22.0 ± 0.6	2.1	11.4

The total number observed is equal to the number of deaths in three independent experimental animals plus the number that was censored. Animals were crawled off the plates, bagged, or bursting and were therefore excluded from all analyses. ^a^ Control indicates the control group (no additional QBLP was added to the medium). ^b^ Met indicates metformin hydrochloride. The log-rank (Mantel–Cox) test was used for statistical analysis. ** *p* < 0.01, *** *p* < 0.001. Data are the mean ± SEM.

## Data Availability

Several data, which were generated during the current study, are not publicly available, but are available from the corresponding author on reasonable request.

## Supplementary Materials

The following supporting information can be downloaded at https://www.mdpi.com/article/10.3390/nu14193976/s1: Table S1: Primer sequences for real-time RT–qPCR validation of gene expression in *C. elegans* designed with Primer3plus software (www.primer3plus.com (accessed on 6 March 2022)); Table S2: Overview of RNA-Seq data; Table S3: Significantly differentially expressed genes; Table S4: Results of GO analysis; Table S5: Results of KEGG pathway analysis; Table S6: Longevity regulating pathway-worm-related genes that were enriched in *C. elegans*; Table S7: DEGs related to DAF-16 treated by QBLP in *C. elegans*; Table S8: Effect of QBLP supplementation on the lifespans of mutant worms CF1038 (*daf-16 (mu86)*).

## References

[1] Culig L., Chu X., Bohr V.A. (2022). Neurogenesis in aging and age-related neurodegenerative diseases. Ageing Res. Rev..

[2] Kritsilis M., Rizou S.V., Koutsoudaki P.N., Evangelou K., Gorgoulis V.G., Papadopoulos D. (2018). Ageing, Cellular Senescence and Neurodegenerative Disease. Int. J. Mol. Sci..

[3] Nadeeshani H., Li J.Y., Ying T.L., Zhang B.H., Lu J. (2022). Nicotinamide mononucleotide (NMN) as an anti-aging health product-Promises and safety concerns. J. Adv. Res..

[4] (2019). United Nations: Global Issues, Ageing. https://www.un.org/en/sections/issues-depth/ageing/.

[5] Li Z.H., Cheng J., Huang L., Li W.X., Zhao Y.P., Lin W.Y. (2021). Aging Diagnostic Probe for Research on Aging and Evaluation of Anti-aging Drug Efficacy. Anal. Chem..

[6] Dong D., Dong M., Liu K., Yu B. (2018). Antioxidant activity of queen bee larvae processed by enzymatic hydrolysis. J. Food Processing Preserv..

[7] Hartfelder K., Guidugli-Lazzarini K.R., Cervoni M.S., Santos D.E., Humann F.C. (2015). Old threads make new tapestry-rewiring of signalling pathways underlies caste phenotypic plasticity in the honey bee, *Apis mellifera* L.. Adv. Insect Physiol..

[8] Tang Q.H., Xiong J., Wang J.X., Cao Z., Liao S.Q., Xiao Y., Tian W.L., Guo J. (2021). Queen bee larva consumption improves sleep disorder and regulates gut microbiota in mice with PCPA-induced insomnia. Food Biosci..

[9] Haber M., Mishyna M., Martinez J.J.I., Benjamin O. (2019). Edible larvae and pupae of honey bee (*Apis mellifera*): Odor and nutritional characterization as a function of diet. Food Chem..

[10] Wu X.L., He K., Velickovic T.C., Liu Z.G. (2021). Nutritional, functional, and allergenic properties of silkworm pupae. Food Sci. Nutr..

[11] Gao Y., Zhao Y.J., Xu M.L., Shi S.S. (2021). *Clanis bilineata tsingtauica*: A Sustainable Edible Insect Resource. Sustainability.

[12] del Hierro J.N., Gutierrez-Docio A., Otero P., Reglero G., Martin D. (2020). Characterization, antioxidant activity, and inhibitory effect on pancreatic lipase of extracts from the edible insects *Acheta domesticus* and *Tenebrio molitor*. Food Chem..

[13] Hall F., Johnson P.E., Liceaga A. (2018). Effect of enzymatic hydrolysis on bioactive properties and allergenicity of cricket (*Gryllodes sigillatus*) protein. Food Chem..

[14] Cho H.D., Min H.J., Won Y.S., Ahn H.Y., Cho Y.S., Seo K.I. (2019). Solid state fermentation process with *Aspergillus kawachii* enhances the cancer-suppressive potential of silkworm larva in hepatocellular carcinoma cells. BMC Complementary Altern. Med..

[15] Seo M., Goo T.W., Chung M.Y., Baek M., Hwang J.S., Kim M.A., Yun E.Y. (2017). *Tenebrio molitor* Larvae Inhibit Adipogenesis through AMPK and MAPKs Signaling in 3T3-L1 Adipocytes and Obesity in High-Fat Diet-Induced Obese Mice. Int. J. Mol. Sci..

[16] Lacroix I.M.E., Teran I.D., Fogliano V., Wichers H.J. (2019). Investigation into the potential of commercially available lesser mealworm (*A. diaperinus*) protein to serve as sources of peptides with DPP-IV inhibitory activity. Int. J. Food Sci. Technol..

[17] Reigada I., Kapp K., Maynard C., Weinkove D., Valero M.S., Langa E., Hanski L., Gomez-Rincon C. (2022). Alterations in Bacterial Metabolism Contribute to the Lifespan Extension Exerted by Guarana in *Caenorhabditis elegans*. Nutrients.

[18] Liao V.H.C. (2018). Use of *Caenorhabditis elegans* To Study the Potential Bioactivity of Natural Compounds. J. Agric. Food Chem..

[19] Li S.T., Zhao H.Q., Zhang P., Liang C.Y., Zhang Y.P., Hsu A.L., Dong M.Q. (2019). DAF-16 stabilizes the aging transcriptome and is activated in mid-aged *Caenorhabditis elegans* to cope with internal stress. Aging Cell.

[20] Ye Y.L., Gu Q.Y., Sun X.L. (2020). Potential of *Caenorhabditis elegansas* an antiaging evaluation model for dietary phytochemicals: A review. Compr. Rev. Food Sci. Food Saf..

[21] Peng Y., Sun Q.C., Gao R.C., Park Y. (2019). AAK-2 and SKN-1 Are Involved in Chicoric-Acid-Induced Lifespan Extension in *Caenorhabditis elegans*. J. Agric. Food Chem..

[22] Lopez-Garcia G., Cilla A., Barbera R., Genoves S., Martorell P., Alegria A. (2020). Effect of plant sterol and galactooligosaccharides enriched beverages on oxidative stress and longevity in *Caenorhabditis elegans*. J. Funct. Foods.

[23] Deng Y.F., Liu H., Huang Q., Tu L.Y., Hu L., Zheng B.S., Sun H.Q., Lu D.J., Guo C.W., Zhou L. (2022). Mechanism of Longevity Extension of *Caenorhabditis elegans* Induced by *Schizophyllum* commune Fermented Supernatant With Added Radix Puerariae. Front. Nutr..

[24] Li Y., Zhang Y., Gan Q.W., Xu M., Ding X., Tang G.H., Liang J.J., Liu K., Liu X.Z., Wang X. (2018). *C. elegans*-based screen identifies lysosome-damaging alkaloids that induce STAT3-dependent lysosomal cell death. Protein Cell.

[25] Li P., Wang Z.H., Lam S.M., Shui G.H. (2021). Rebaudioside A Enhances Resistance to Oxidative Stress and Extends Lifespan and Healthspan in *Caenorhabditis elegans*. Antioxidants.

[26] Fang E.F., Waltz T.B., Kassahun H., Lu Q.P., Kerr J.S., Morevati M., Fivenson E.M., Wollman B.N., Marosi K., Wilson M.A. (2017). Tomatidine enhances lifespan and healthspan in *C. elegans* through mitophagy induction via the SKN-1/Nrf2 pathway. Sci. Rep..

[27] Bansal A., Zhu L.H.J., Yen K., Tissenbaum H.A. (2015). Uncoupling lifespan and healthspan in *Caenorhabditis elegans* longevity mutants. Proc. Natl. Acad. Sci. USA.

[28] Serna E., Mastaloudis A., Martorell P., Wood S.M., Hester S.N., Bartlett M., Prolla T.A., Vina J. (2020). A Novel Micronutrient Blend Mimics Calorie Restriction Transcriptomics in Multiple Tissues of Mice and Increases Lifespan and Mobility in *C. elegans*. Nutrients.

[29] Park S., Kim B.K., Park S.K. (2021). Supplementation with phosphatidylethanolamine confers anti-oxidant and anti-aging effects via hormesis and reduced insulin/IGF-1-like signaling in *C. elegans*. Mech. Ageing Dev..

[30] Kim H.M., Long N.P., Yoon S.J., Nguyen H.T., Kwon S.W. (2019). Metabolomics and phenotype assessment reveal cellular toxicity of triclosan in *Caenorhabditis elegans*. Chemosphere.

[31] Machalinski B., Roginska D., Wilk A., Szumilas K., Prowans P., Paczkowska E., Szumilas P., Stecewicz I., Zawodny P., Zietek M. (2021). Global Gene Expression of Cultured Human Dermal Fibroblasts: Focus on Cell Cycle and Proliferation Status in Improving the Condition of Face Skin. Int. J. Med. Sci..

[32] Guha S., Cao M., Kane R.M., Savino A.M., Zou S.G., Dong Y.Q. (2013). The longevity effect of cranberry extract in *Caenorhabditis elegans* is modulated by *daf-16* and *osr-1*. Age.

[33] Wang X.X., Cook L.F., Grasso L.M., Cao M., Dong Y.Q. (2015). Royal jelly-mediated prolongevity and stress resistance in *Caenorhabditis elegans* is possibly modulated by the interplays of DAF-16, SIR-2.1, HCF-1, and 14-3-3 proteins. J. Gerontol. Biol. Sci..

[34] Chen X.L., Lui E.Y., Ip Y.K., Lam S.H. (2018). RNA sequencing, de novo assembly and differential analysis of the gill transcriptome of freshwater climbing perch Anabas testudineus after 6 days of seawater exposure. J. Fish Biol..

[35] Herndon L.A., Schmeissner P.J., Dudaronek J.M., Brown P.A., Listner K.M., Sakano Y., Paupard M.C., Hall D.H., Driscoll M. (2002). Stochastic and genetic factors influence tissue-specific decline in aging *C. elegans*. Nature.

[36] Sun N., Youle R.J., Finkel T. (2016). The Mitochondrial Basis of Aging. Mol. Cell.

[37] O’Rourke E.J., Kuballa P., Xavier R., Ruvkun G. (2013). omega-6 Polyunsaturated fatty acids extend life span through the activation of autophagy. Genes Dev..

[38] Wang C., Li Y.Y., Zeng L.J., Shi C.L., Peng Y., Li H., Chen H.B., Yu J., Zhang J., Cheng B. (2022). Tris(1,3-dichloro-2-propyl) phosphate reduces longevity through a specific microRNA-mediated DAF-16/FoxO in an unconventional insulin/ insulin-like growth factor-1 signaling pathway. J. Hazard. Mater..

[39] Scerbak C., Vayndorf E., Hernandez A., McGill C., Taylor B. (2018). Lowbush cranberry acts through DAF-16/FOXO signaling to promote increased lifespan and axon branching in aging posterior touch receptor neurons. Geroscience.

[40] Chen Q., Xu B.J., Huang W.S., Amrouche A.T., Maurizio B., Simal-Gandara J., Tundis R., Xiao J.B., Zou L., Lu B.Y. (2020). Edible flowers as functional raw materials: A review on anti-aging properties. Trends Food Sci. Technol..

[41] Luyten W., Antal P., Braeckman B.P., Bundy J., Cirulli F., Fang-Yen C., Fuellen G., Leroi A., Liu Q., Martorell P. (2016). Ageing with elegans: A research proposal to map healthspan pathways. Biogerontology.

[42] Shen P.Y., Yue Y.R., Zheng J., Park Y. (2018). *Caenorhabditis elegans*: A Convenient In Vivo Model for Assessing the Impact of Food Bioactive Compounds on Obesity, Aging, and Alzheimer’s Disease. Annu. Rev. Food Sci. Technol..

[43] Zheng S.Q., Liao S.T., Zou Y.X., Qu Z., Liu F. (2014). *ins-7* Gene Expression Is Partially Regulated by the DAF-16/IIS Signaling Pathway in *Caenorhabditis elegans* under Celecoxib Intervention. PLoS ONE.

[44] Zhou L., Liu J., Bu L.L., Liao D.F., Cheng S.W., Zheng X.L. (2021). Curcumin Acetylsalicylate Extends the Lifespan of *Caenorhabditis elegans*. Molecules.

[45] Sugawara T., Sakamoto K. (2018). Killed Bifidobacterium longum enhanced stress tolerance and prolonged life span of *Caenorhabditis elegans* via DAF-16. Br. J. Nutr..

[46] Chen S., Whetstine J.R., Ghosh S., Hanover J.A., Gali R.R., Grosu P., Shi Y. (2009). The conserved NAD(H)-dependent corepressor CTBP-1 regulates *Caenorhabditis elegans* life span. Proc. Natl. Acad. Sci. USA.

[47] Elle I.C., Olsen L.C.B., Pultz D., Rodkaer S.V., Faergeman N.J. (2010). Something worth dying for: Molecular tools for the dissection of lipid metabolism in *Caenorhabditis elegans*. FEBS Lett..

[48] Hansen M., Flatt T., Aguilaniu H. (2013). Reproduction, Fat Metabolism, and Life Span: What Is the Connection?. Cell Metab..

[49] McCormick M., Chen K., Ramaswamy P., Kenyon C. (2012). New genes that extend *Caenorhabditis elegans*’ lifespan in response to reproductive signals. Aging Cell.

[50] Matsuoka T., Kawashima T., Nakamura T., Kanamaru Y., Yabe T. (2012). Isolation and characterization of proteases that hydrolyze royal jelly proteins from queen bee larvae of the honeybee, Apis mellifera. Apidologie.

[51] Wang X.X., Cao M., Dong Y.Q. (2016). Royal jelly promotes DAF-16-mediated proteostasis to tolerate β-amyloid toxicity in *C. elegans* model of Alzheimer’s disease. Oncotarget.

[52] Sun Y.N., Li M.J., Zhao D.F., Li X., Yang C.L., Wang X.C. (2020). Lysosome activity is modulated by multiple longevity pathways and is important for lifespan extension in *C. elegans*. eLife.

[53] Qian H., Xu X.R., Niklason L.E. (2015). Bmk-1 regulates lifespan in *Caenorhabditis elegans* by activating hsp-16. Oncotarget.

[54] Hars E.S., Qi H.Y., Ryazanov A.G., Jin S.K., Cai L., Hu C.C., Liu L.F. (2007). Autophagy regulates ageing in C-elegans. Autophagy.

