# Supplementary material for: Supplementation with Queen Bee Larva Powder Extended the Longevity of Caenorhabditis elegans
Source: Nutrients. 2022 Sep 24;14(19):3976. doi: 10.3390/nu14193976 (PMC9573043; doi:10.3390/nu14193976)
Supplement: Supplementary file 1 [file nutrients-14-03976-s001.zip › Supplementary Table S1.pdf]

Supplementary Table S1. Primer sequences for real-time RT–qPCR validation of gene expression in *C. elegans* designed with Primer3plus software (www.primer3plus.com).

Gene	Primer sequence (5′ to 3′)		Product length (bp)	PCR Cycles
dpy-5	forward	ctgagcaatacgttcgtgga	21	40
	reverse	tccagttggtccgttctttc	21	40
dpy-13	forward	cgtgctttcggtttgcattact	22	40
	reverse	ttcatggatcccttgcaggtaag	23	40
col-107	forward	ccatcgttattggagcgtctttc	23	40
	reverse	cggtgtttctgaactcagtcatg	23	40
dct-8	forward	ctcattgttctcctcggccttat	23	40
	reverse	gtaggctccctggtttccatatc	23	40
dod-24	forward	cttcttcgtcaaaccgcaat	20	40
	reverse	tgtgagagacttccgatgtagg	22	40
dct-16	forward	atcgccgccacttacaagaa	20	40
	reverse	cggcttccgattgtccctta	20	40
dct-7	forward	aatcggactcaacactggattgg	23	40
	reverse	ctccaacaagtcctcctccaac	22	40
