# Supplementary material for: Supplementation with Queen Bee Larva Powder Extended the Longevity of Caenorhabditis elegans
Source: Nutrients. 2022 Sep 24;14(19):3976. doi: 10.3390/nu14193976 (PMC9573043; doi:10.3390/nu14193976)
Supplement: Supplementary file 1 [file nutrients-14-03976-s001.zip › Supplementary Table S2.pdf]

Table S2. Overview of RNA-Seq data.

Sample	Raw reads	Clean reads	Error rate (%)	Total mapped (%)	Multiple mapped (%)	Uniquely mapped (%)
S_14_3	47716312	45854876	0.0259	96.73	1.98	94.75
S_14_2	53119192	51084886	0.0259	96.49	1.88	94.61
S_14_1	47946994	46474184	0.0252	97.12	1.96	95.15
C_14_3	49774900	48089178	0.0258	96.60	1.87	94.73
C_14_2	49413888	48655400	0.0258	96.58	1.88	94.7
C_14_1	50876092	49277158	0.0253	96.52	1.87	94.64
