# Supplementary material for: Supplementation with Queen Bee Larva Powder Extended the Longevity of Caenorhabditis elegans
Source: Nutrients. 2022 Sep 24;14(19):3976. doi: 10.3390/nu14193976 (PMC9573043; doi:10.3390/nu14193976)
Supplement: Supplementary file 1 [file nutrients-14-03976-s001.zip › Supplementary Table S6.pdf]

Supplementary Table S6. Longevity regulating pathway-worm-related genes that were enriched in *C. elegans*.

Gene_id	Gene name	Gene_description	Fold change	*p*-value	Significant	Regulate
WBGene00004932	*sod-3*	Superoxide dismutase [Mn] 2, mitochondrial	2.087	0.00	yes	up
WBGene00001754	*gst-6*	Probable glutathione S-transferase 6	2.204	0.00	yes	up
WBGene00002013	*hsp-12.6*	Heat Shock Protein; Heat shock protein 12.6	3.126	0.00	yes	up
WBGene00015645	*lips-7*	LIPaSe related	4.013	0.01	yes	up
WBGene00002091	*ins-8*	INSulin related	2.857	0.00	yes	up
WBGene00019939	*lips-17*	LIPaSe related	83.982	0.00	yes	up
