# Supplementary material for: Supplementation with Queen Bee Larva Powder Extended the Longevity of Caenorhabditis elegans
Source: Nutrients. 2022 Sep 24;14(19):3976. doi: 10.3390/nu14193976 (PMC9573043; doi:10.3390/nu14193976)
Supplement: Supplementary file 1 [file nutrients-14-03976-s001.zip › Supplementary Table S7.pdf]

Supplementary Table S7. DEGs related to DAF-16 treated by QBLP in *C. elegans*.

Gene_id	Gene name	Gene_description	Fold change	*p*-value	Significant	Regulate
WBGene00010125	*dod-22*	Downstream Of DAF-16 (Regulated by DAF-16)	10.94	0.00	yes	up
WBGene00010745	*dod-17*	Downstream Of DAF-16 (Regulated by DAF-16)	5.25	0.00	yes	up
WBGene00044472	*dct-8*	DAF-16/FOXO Controlled, germline Tumor affecting	4.09	0.00	yes	up
WBGene00007875	*dod-24*	Downstream Of DAF-16 (Regulated by DAF-16)	4.08	0.00	yes	up
WBGene00016052	*dod-3*	Downstream Of DAF-16 (Regulated by DAF-16)	2.79	0.00	yes	up
WBGene00012615	*dct-16*	DAF-16/FOXO Controlled, germline Tumor affecting	2.65	0.00	yes	up
WBGene00017488	*dct-7*	DAF-16/FOXO Controlled, germline Tumor affecting	2.59	0.00	yes	up
