# Supplementary material for: Supplementation with Queen Bee Larva Powder Extended the Longevity of Caenorhabditis elegans
Source: Nutrients. 2022 Sep 24;14(19):3976. doi: 10.3390/nu14193976 (PMC9573043; doi:10.3390/nu14193976)
Supplement: Supplementary file 1 [file nutrients-14-03976-s001.zip › Supplementary Table S8.pdf]

Supplementary Table S8. Effect of QBLP supplementation on the lifespans of mutant worms CF1038 (*daf-16 (mu86)*).

Condition	Number of animals that died /total	Mean lifespan (days ± SEM)	Median lifespan (days ± SEM)	Change from control (d)	Increase versus control (%)
Control[a]	117/120	12.4±0.2	12.0±0.2	/	/
0.2 g/L	114/120	12.0±0.2	12.0±0.2	-0.4	-3.2

The total number observed is equal to the number of deaths in three independent experimental animals plus the number that was censored. Animals were crawled off the plates, bagged, or bursting and were therefore excluded from all analyses. [a] Control indicates the control group (no additional QBLP was added to the medium). The log-rank (Mantel-Cox) test was used for statistical analysis. Data are the mean ± SEM.
